# The content of patient counseling about interchangeable medicines and generic substitution in Finnish community pharmacies - a survey of dispensers

**DOI:** 10.1186/s12913-019-4798-2

**Published:** 2019-12-11

**Authors:** Riikka Rainio, Riitta Ahonen, Johanna Timonen

**Affiliations:** School of Pharmacy/ Social Pharmacy, Faculty of Health Sciences, Kuopio Campus, University of Eastern Finland, Box 1627, FI-70211 Kuopio, PO Finland

**Keywords:** Generic substitution, Interchangeable medicines, Reference price system, Patient counseling, Community pharmacy, Survey

## Abstract

**Background:**

Generic substitution aims to increase the use of more affordable generic preparations and restrain the growth of medicine expenditures. Pharmaceutical staff plays an important role in generic substitution by implementing substitution and counseling customers. The aim of this study was to explore how Finnish dispensers inform pharmacy customers about interchangeable medicines and generic substitution and what customers ask dispensers about generic substitution and the reference price system.

**Methods:**

A questionnaire was sent to a random sample of dispensers (*n* = 1054) working in community pharmacies in spring 2018. The data was analyzed using frequencies, percentages and the Chi-square test and Fisher’s exact test. The open-ended questions were analyzed first using inductive content analysis and later with the quantitative methods mentioned above.

**Results:**

The final study material consisted of 498 questionnaires (response rate 51%). The main topics dispensers always informed customers about were the physician’s record in the prescription not to substitute the medicine (69%) and that the prescribed or purchased interchangeable medicine did not belong in the reference price band (59%). Topics mentioned often by dispensers dealt with customer’s chance to choose their medicine from among several alternative products (68%) and the manufacturer of the interchangeable medicines (66%). Differences in appearance (33%) or in composition (28%) of interchangeable products were the most common topics mentioned only when the customer asked about them. Of the respondents 17.6% always and 51.4% often informed the customer about the least expensive interchangeable product at the point of dispensing. Customers’ questions about the generic substitution and reference price system most commonly (82.4%) concerned the similarity of interchangeable medicines.

**Conclusions:**

Finnish dispensers provide customers with a wide range of information about different subjects when dispensing interchangeable medicines. Patient counseling generally meets the legislative requirements, except for price counseling. In future, information about generic substitution and interchangeable medicines should continuously be provided to customers both at pharmacies and elsewhere, e.g. through educational campaigns.

## Background

Escalating pharmaceutical expenditures and their reimbursement costs are a global concern in healthcare [1]. New, more expensive medicines and aging populations are two of the main reasons for this [2]. Generic substitution (GS) and reference price system (RPS) have been important in attempts to restrain the growth of pharmaceutical expenditures [2, 3]. They curb expenditures by promoting the use of more affordable generic preparations and stimulating price competition between interchangeable medicines. GS is already widely used around the globe including in 41 European countries, the United States, Canada and Australia [3–6], and substantial cost savings have been achieved.

According to several studies, pharmaceutical staff play a significant role in GS by implementing substitution and counseling customers [7–13]. Customers who have received information about substitution and interchangeable medicines have a more positive view about these matters [8, 12, 14–17]. Conversely, lack of information is associated with confusion, uncertainty and negative attitudes towards substitution [8, 12, 15, 18–21]. It may also reduce patient compliance [18, 20] and threaten medication safety, e.g. in cases where one interchangeable preparation gets confused with another [18, 22]. However, only a few studies have addressed the content of patient counseling about GS and interchangeable medicinal products [23–25].

In Finland, mandatory GS was adopted at the beginning of April 2003 [26, 27]. Since then pharmaceutical staff have been obliged to substitute a prescribed medicine with the cheapest or close-to-cheapest interchangeable medicine, unless either physician or customer prohibits this [28]. GS in pharmacies is based on the list of interchangeable medicines created by the Finnish Medicines Agency Fimea. Medicines are considered interchangeable when they contain the same quantity of the same active substance, are biologically equivalent and have the same pharmaceutical form [29]. There are certain exceptions to the list, e.g. tablets and capsules may be substituted for each other, and for pharmacological or clinical reasons some medicine groups are not included in the scope of GS.

At the beginning of April 2009, GS was supplemented with the RPS [27], in which interchangeable medicines are clustered into reference price groups and for each group a reference price is defined [30]. The reference price sets a limit on the price for which reimbursement is available and is computed by adding €0.50 to the retail price of the least expensive interchangeable product in the reference price group. This €0.50 price difference is also called the reference price band, and medicines belonging in the price band are reimbursed based on their retail price. If the medicine is priced higher than the reference price and the customer does not want to substitute the medicine for an interchangeable medicine within the same price band, he or she must pay the excess in addition to the co-payment. Reference price groups, products included in the groups and reference prices are determined every three months. However, pharmaceutical companies can change the pricing of their products every two weeks.

In Finland GS and RPS have resulted in significant cost savings. By the beginning of 2018 they had generated savings of over €1 billion, of which around €850 million were savings for medicine users and nearly €150 million were savings for the Health Insurance Scheme [31]. Even though GS and RPS mean cost savings, both the customer and the physician can prohibit substitution. In 2017 around 41.8 million reimbursable medicine prescriptions were purchased, of which around 31.1 million (74.4%) prescriptions were eligible for GS [32]. Of these, customers declined substitution in 4.9% of cases and physicians in 1.2%.

In Finland, only pharmacists (M.Sc. in Pharmacy) and dispensers (B.Sc. in Pharmacy) are allowed to dispense prescriptions and counsel customers about medicines [28]. Pharmacies are required to counsel customers about the correct and safe use of medicines. Customers must also be given information about medicine prices and other factors affecting their choice of product. Recently many legislative changes to GS and RPS have been introduced in order to reduce the prices of interchangeable medicines and promote their use and price competition [33, 34]. These changes have altered the principles of GS and set new requirements for patient counseling in pharmacies. Since 2016 price counseling in pharmacies about prescription medicines has had to include information about the least expensive interchangeable product at the point of dispensing [33, 35] In addition, at the beginning of 2017 the reference price band was narrowed from €1.50–€2.00 down to €0.50 [34, 36].

The aim of this study was to explore (1) what information Finnish dispensers provide to pharmacy customers about interchangeable medicinal products and GS, (2) whether counseling includes information about the least expensive interchangeable product, and (3) what kind of questions pharmacy customers ask dispensers about GS and the RPS.

## Methods

### Study setting

The cross-sectional postal survey was conducted in February and March 2018. The questionnaire was sent to a random sample (one–third) of dispensers (*n* = 1054) working in community pharmacies. Dispensers (B.Sc. in Pharmacy) were chosen as the target group because they are the biggest occupational group in pharmacies and are mainly responsible for dispensing prescriptions and patient counseling, whereas pharmacists (M.Sc. in Pharmacy) usually work as managers alongside the pharmacy owner (M.Sc. in Pharmacy) [37]. The sample was obtained from the register of the Finnish Pharmacists’ Association. A reminder was mailed twice to each recipient. The response period was approximately two weeks in all mailing rounds.

The four-page questionnaire consisted of 18 questions (Additional file 1). The questions were designed on the basis of the legislative requirements set for the content of pharmacy customer counseling about GS [28, 38] and some previous studies [7, 9, 11, 39, 40]. The face validity of the questionnaire was tested by all the authors and by five research colleagues familiar with questionnaires as a research method and the study concept. The questionnaire was piloted simultaneously in two pharmacies, where dispensers filled in the questionnaire and commented on the intelligibility of the questions. Minor modifications were made to the questionnaire on the basis of the pilot.

This paper examines the responses to the four questions concerning the content of dispenser-customer communication. The content of pharmacy customer counseling was investigated with the question: “What do you tell customers about medicines included in the RPS when dispensing prescriptions?”. The question offered 15 fixed responses. Respondents were instructed to answer the question using four response options to indicate how often they discussed the listed topics with customers: 1 = always, 2 = often, 3 = rarely, 4 = only when asked about it. The respondents also were able to list other commonly discussed topics with space for a freely worded answer. In order to investigate whether counseling includes information about the least expensive interchangeable product, the respondents were asked whether they provide this information at the point of dispensing RPS medicines. The question had the same four response options as the question mentioned earlier. If the respondents answered using options 2 = often, 3 = rarely or 4 = only when asked about it, they were also asked to clarify the situations in which they do not tell customers about the least expensive interchangeable product. In addition, dispensers were asked with open-ended question about the questions customers have about GS and the RPS.

Background information on the respondents was collected with an open-ended question about age and structured questions about gender, the location of the pharmacy and the number of prescriptions purchased per year in the pharmacy. At the beginning of the questionnaire the respondents were asked to state their current job at the pharmacy. Respondents not currently working in a pharmacy were asked to return the questionnaire blank.

The study setting and research process were in accordance with local and national ethical instructions for research [41–43]. This study required no ethical approval.

### Data analysis

The data was analyzed using IBM SPSS Statistics for Windows, Version 25 using frequencies, percentages and the Chi-square test and Fisher’s exact test. The Chi-square test or Fisher’s exact test was used to compare the representativeness of the study population with respect to age and gender, and to examine whether either of these factors was associated with how they answered the question about informing customers about the least expensive interchangeable product. The open-ended questions were analyzed first using inductive content analysis and later with the quantitative methods mentioned above. The level of statistical significance was defined as *p*-values < 0.05.

## Results

After two reminders a total of 572 questionnaires were returned. However, 74 questionnaires were excluded because the respondents stated they did not currently work in a community pharmacy (*n* = 66) or returned the questionnaire blank (*n* = 8). Consequently, the final study sample was 980 dispensers of whom 498 (51%) returned the completed questionnaire. The representativeness of the study population was analyzed with respect to age and gender, and was found to be largely representative of the target population. However, dispensers aged ≤29 years were overrepresented in the study sample compared to the target population (15.5% versus 11.4%, *p*-value = 0.009) (Table 1).

**Table 1:** Characteristics and representativeness^a^ of the study population

	Respondent dispensers % (n)	Target dispensers % (n)^b^
*Gender*	*n* = 493^c^	*n* = 3253
Female	94.5 (466)	95.1 (3095)
Male	5.5 (27)	4.9 (158)
*Age, years*	*n* = 496^c^	n = 3253
≤ 29	15.5 (77) *	11.4 (372)*
30–39	26.8 (133)	27.3 (889)
40–49	26.8 (133)	30.6 (994)
50–59	24.4 (121)	25.9 (841)
≥ 60	6.5 (32)	4.8 (157)
*Number of prescriptions per year at the pharmacy*	n = 493^c^	
≤ 30,000	7.7 (38)	
30,001–60,000	15.4 (76)	
60,001–100,000	31.2 (154)	
≥ 100,001	45.6 (225)	
*Location of the pharmacy*	*n* = 492^c^	
Southern Finland	30.1 (148)	
Southwest Finland	11.8 (58)	
Western and Inland Finland	26.4 (130)	
Eastern Finland	17.9 (88)	
Northern Finland	9.8 (48)	
Lapland	4.1 (20)	

^a^Representativeness was analyzed by the respondent’s age and gender^b^Information based on the register of the Finnish Pharmacists’ Association in January 2018^c^Some of the respondents did not report their gender, age, number of prescriptions per year at the pharmacy or the location of the pharmacy

^a^*p*-value = 0.009, Chi-square test

### Dispenser-customer communication during the interchangeable medicine prescription purchase

**Fig. 1:**
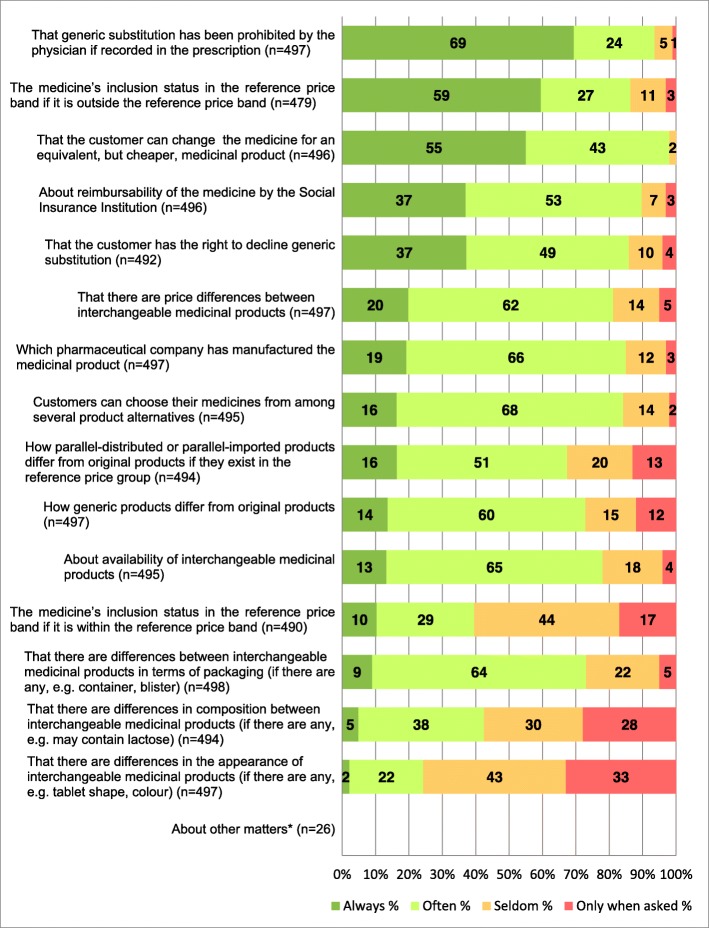
Information provided by dispensers to customers about the interchangeable medicines included in the reference price system. ^a^ e.g. information about reference price system, possible differences in storage conditions, the preparation the customer used last time, the interchangeable medicines contain the same active substance.

Dispensers provided a wide range of information about different topics when dispensing interchangeable medicinal products included in the RPS. Most of the topics were discussed often or always (Fig. 1). Those that were always mentioned to customers were the physician’s record in the prescription not to substitute the medicine (69%), the notion that the prescribed or purchased interchangeable medicine did not belong in the reference price band (59%), and the customer’s option to substitute the medicine for an equivalent but cheaper medicinal product (55%). Often-mentioned topics dealt with the customer’s right to choose their medicine from among several alternative products (68%), the manufacturer of the interchangeable medicinal product (66%), and the availability of the interchangeable medicinal products (65%). Differences in appearance (33%) or in composition (28%) of interchangeable products were the most common pieces of information given only when customers asked about them. The same information, together with the medicine’s inclusion status in the reference price band for products within the price band, was mentioned least often (Fig. 1).

### Price counseling during the dispenser-customer communication

Of the respondents 17.6% (*n* = 86) always informed the customer about the least expensive interchangeable product at the point of dispensing. Over half (51.4%, *n* = 251) stated they often inform the customer about the least expensive product, whereas around one-third informed customer rarely (26.0%, *n* = 127) or only when the customer asks about it (4.9%, *n* = 24). Men were more likely than women to inform the customer about the least expensive interchangeable product only when the customer asks about it (14.8% *n* = 4 versus 4.4% *n* = 20, *p*-value = 0.038). There were no statistically significant differences with respect to respondent’s age.

Respondents who did not always inform the customer about the least expensive interchangeable product were asked to clarify the situations in which they do not do so. The three most frequently given reasons were the small price difference between the least expensive and other interchangeable products (68.3%), the unavailability of the least expensive product in the pharmacy or from the pharmaceutical wholesaler (51.9%), and the customer’s earlier or long-term use of a specific interchangeable product (26.7%) (Table 2).

**Table 2:** Reasons why the dispenser does not inform the customer about the least expensive interchangeable product at the point of dispensing (*n* = 397)

Factors	Percent of cases % (n) ^a^
Small price difference between the least expensive and other interchangeable medicinal products	68.3 (271)
The least expensive interchangeable product is not available at the community pharmacy or from the pharmaceutical wholesaler	51.9 (206)
The customer has been using the same preparation for a shorter or longer period of time	26.7 (106)
Attributable to the customer (difficulty understanding the substitution, memory disorder, possible risk of confusing the substituted preparations)	16.6 (66)
The customer has previously declined the substitution, or the customer wishes to have the same preparation as before or the one prescribed by his/her physician	13.4 (53)
There are other interchangeable products available in the reference price band	4.3 (17)
The customer needs to have the medicine at once (e.g. antibiotics)	3.8 (15)
Work pressure in the pharmacy	2.5 (10)
The person purchasing the prescription is not the user of the medicine	2.5 (10)
Substitution does not affect the cost to the customer (e.g. full reimbursement, private insurance company, social assistance certificate)	2.5 (10)
Other^b^	10.3 (41)

^a^respondents could give several reasons in the situation

^b^e.g. the customer does not ask for the cheapest product, the customer cannot get reimbursement for the cheapest product, medicine prices/reference price band has changed recently

### Questions about GS and RPS

The most common questions customers ask dispensers about the GS and the RPS concerned the similarity of interchangeable medicinal products (Table 3). The other commonly asked questions dealt with the prices and differences between interchangeable medicinal products. Questions directly about the RPS were not common.

**Table 3:** Questions customers usually present about generic substitution and reference price system (*n* = 465)

Themes of the questions	Examples of the most commonly asked questions of the theme	Percent of cases % (n) ^a^
The similarity and equivalence of interchangeable medicinal products (similarity, effectiveness, effect, strength, equally good)	“Is it the same medicine?” “Are the medicines really alike?” “Is it as effective as the previous one?” “Do they act in the same way?” “Are the medicines equally good?” “Do they have the same amount of active ingredient?”	82.4 (383)
The price of the interchangeable medicinal products (price difference, frequent changing of prices)	“What is the price difference?” “How much does the cheaper one cost?” “How come it can be so much cheaper/more expensive?” “Why do the prices vary?” “How come the price is again different compared to last time?”	34.6 (161)
Differences between interchangeable medicinal products	“How do the interchangeable medicines differ from each other?” “What is the difference?”	20.0 (93)
Side effects and the suitability of the interchangeable medicinal products	“Does it have more/different side effects?” “Is the new drug really suitable for me?” “Is it really safe to change the medicine?” “Do I dare to change my medicine?”	8.2 (38)
Reimbursements for medicine expenses	“How come I can’t get the full reimbursement when I choose the more expensive one?”	7.3 (34)
The origin of the interchangeable medicinal products	“Where has the drug been made?” “Who is the manufacturer?” “Is it a Finnish product?”	7.1 (33)
The cheaper/cheapest interchangeable medicinal product	“What is the cheapest product?” “Do you have a cheaper one?”	4.9 (23)
Properties of the interchangeable medicinal products (excipients, dividing, appearance, package)	“Does it contain lactose” “Are the tablets scored?” “Can the tablets be split in half?” “How big is the tablet?” “Are the tablets in a blister pack or in a bottle?”	4.7 (22)
Generic substitution in practice	“Must I really change my medicine?” “Can I choose differently next time?”	4.5 (21)
Physician and the generic substitution	“Do I need a physician’s permission to change my medicine?” “Should I first ask my physician?”	3.2 (15)
Reference price system	“What is the reference price system?” “What does the reference price mean?”	3.2 (15)
Other	“Why don’t you have the cheapest product in stock?” “Why did the physician choose to prescribe this product?” “Why are there so many different options for the same medicine?” “Why do you have to ask me to change my medicine when the price difference is so small?”	7.1 (33)

^a^respondents could list several questions

## Discussion

According to this study, Finnish dispensers inform customers about many different topics when dispensing interchangeable medicinal products included in the RPS. Patient counseling about interchangeable medicines and GS generally meets the legislative requirements, except for price counseling. Even though price differences between interchangeable medicines are often discussed with customers, only one-fifth of the Finnish dispensers always inform customers about the price differences. In addition, information about the least expensive interchangeable product is not always included in the counseling.

This study shows that dispensers in Finland use their professional judgement when not informing customers about the least expensive interchangeable product. The most common reasons for not informing the customer were the small price difference between the least expensive and other interchangeable products, and the unavailability of the least expensive product in the pharmacy or from the pharmaceutical wholesaler. It might be inconvenient to inform the customer about the least expensive interchangeable product if the price difference is just a couple of cents, or if the product is not available and the customer needs to have the medicine at once. Similar reasons for not performing or recommending GS have been found in a Japanese study [44].

However, in this study the questions about price differences and changed prices were customers’ second most common questions to dispensers. This indicates that customers are interested in prices and they wish to be given information about them. In many previous studies cost savings have been reported as an important reason why customers and patients substitute their medicine [7, 9, 12, 21, 45–47] . Cost savings for customers and patients have also been recognized as a reason for pharmacists to recommend GS [23, 24, 48] . Hence, price difference can be seen as an important factor affecting the substitution process. Price difference, however, is a subjective matter. From the customer’s point of view, the dispenser is unable to evaluate the customer’s desire to substitute their medicine without asking. This highlights the importance of price counseling during GS.

Another reason for not offering the least expensive interchangeable product concerned the customers themselves, e.g. difficulty in understanding the substitution or the risk of confusion about the substituted preparations. This kind of concern about confused patients due to GS has been recognized among pharmacists in previous studies [23, 49–51]; in addition to our study its significance for substitution has been found in an Australian study [23]. From the customers’ and patients’ point of view*,* confusing factors have been associated with differences in the appearance or packaging of interchangeable medicines [8, 20, 52]. Hence, information about differences between interchangeable medicines might reduce such confusion. Finnish dispensers often informed customers about differences in the packaging of generic medicines. However, differences in appearance were seldom discussed or only when the customer asked about them. This may be due to the difficulty of properly comparing the properties of interchangeable medicines. Even though Finnish pharmacies have databases containing summaries of product characteristics (SPC), the information must be sought separately and the number of alternatives makes the comparison even harder. To prevent confusion dispensers should give customers more information about any changes in the appearance of the medicines they are purchasing. In addition, measures should be taken to facilitate comparison of the properties of interchangeable medicines in order to improve the quality of patient counseling.

The questions customers most commonly asked dispensers about GS and RPS mainly dealt with the similarity of interchangeable medicines. This may indicate customers’ lack of knowledge about interchangeability and substitution policies. The few questions about the practical implementation of GS and the physician’s role in GS support this observation. The findings are significant, because even though GS has been in use in Finland since 2003, it still seems that customers are not always aware of the system. On the other hand, this is to be expected since because of the ageing population there are new medicine users who may not yet be familiar with the system. The questions about similarity may also indicate customers’ mistrust in interchangeable medicines. In previous studies customers and patients have been reported to believe generics to be less effective [13, 14, 21, 47, 53], of inferior quality [15, 20, 53–55] or to cause more side effects [8, 13, 14, 18, 21, 22, 56]. Questions about similarity and side effects were mentioned in our study, too. Patients’ lack of knowledge about substitution and mistrust in generic medicines may function as a barrier to GS and thus undermine the potential cost savings of the system. Hence, it is important constantly to inform medicine users about GS and what interchangeability means and not simply to rely on the information given during the early years of GS. In future, educational campaigns or information material should be provided.

### Strengths and limitations

This study has several strengths. It provides new information about the mandatory GS and RPS with an obligation to provide price counseling. In Finland, the GS and RPS have been in use since 2003 and 2009, respectively, hence dispensers are well experienced in answering the questions. The reasons given in the situation in which dispensers do not inform the customer about the least expensive interchangeable product were obtained through an open-ended question. This provided a broad view of the factors behind the scene.

The respondents were obtained from the register of the Finnish Pharmacists’ Association, which encompasses almost every dispenser in Finland. The response rate of 51% is good and similar to that obtained in the previous generic substitution postal survey for dispensers conducted in Finland in 2004 (54%) [39]. In postal surveys about generic substitution conducted elsewhere the response rate of pharmacists has varied between 15 and 58% [57]. In our study the respondents represented the target population quite well, except that dispensers aged ≤29 years were slightly over represented. Therefore, we suggest that the results can be generalized with caution to apply to all Finnish community dispensers.

Besides background questions, the questions examined in this article have not been used in any previous studies and thus the validity and reliability of these questions have not been tested. However, legislation and some previous studies were utilized when developing the questions. In addition, the face validity of the questionnaire was tested and the questionnaire was pilot tested in two pharmacies. Furthermore, the response rate for the questions posed in this paper was also high (93–99.6%) and thus it can be assumed that the questions were probably understandable. This makes the results more reliable.

It must be noted that the results of this paper are based on dispensers’ self-reports, and thus some aspects of the results may be over- or underemphasized. The results relating to customers’ most common questions about GS and RPS represent dispensers’ own subjective views. This means that the results may have differed if the question had been presented to customers. In future the same question should be put to customers in order to obtain a more comprehensive picture. In addition, the content of counseling was measured using four response options about how often they discussed the listed topics with customers: 1 = always, 2 = often, 3 = rarely, 4 = only when asked about it. Although this gives a broad view about generally discussed subjects, it does not reveal the details of the discussion. With a different research method like an interview or observation study, the content of counseling could be examined in more detail. Although the principles of GS and RPS vary between different countries, the findings of this paper can be utilized provided the differences between systems are taken into account.

## Conclusions

Patient counseling about interchangeable medicines and GS in Finland is generally given according to the legislation. However, price counseling does not entirely fulfill its legislative requirements, because price counseling and information about the least expensive interchangeable medicines is not always given to customers. Finnish dispensers give customers wide-ranging information about different subjects when dispensing interchangeable medicines. According to Finnish dispensers, pharmacy customers’ questions about GS and RPS mainly concern the similarity of interchangeable medicines. This may indicate customers’ inadequate knowledge of the interchangeable medicines and substitution policies. In future, information about GS and interchangeable medicines should continuously be provided to customers both at pharmacies and elsewhere, e.g. through educational campaigns.

## Supplementary information

**Additional file 1.** Questionnaire - Survey for dispensers regarding generic substitution and reference price system.

## Abbreviations

GS: Generic substitution
RPS: Reference price system

## References

[1] Belloni A, Morgan D, Paris V. Pharmaceutical Expenditure And Policies: Past Trends And Future Challenges. OECD Health Working Paper No. 87; 2016 OECD Publishing, Paris. doi: 10.1787/5jm0q1f4cdq7-en

[2] IMS Health. The role of generic medicines in sustaining healthcare systems: a European perspective. IMS Institute for Healthcare Informatics 2015. https://www.iqvia.com/-/media/iqvia/pdfs/institute-reports/the-role-of-generic-medicines-in-sustaining-healthcare-systems.pdf . Accessed 2 Jun 2019

[3] World Health Organization (WHO). Medicines reimbursement policies in Europe 2018. 2018 http://www.euro.who.int/__data/assets/pdf_file/0011/376625/pharmaceutical-reimbursement-eng.pdf . Accessed 2 Jun 2019

[4] Song Y, Barthold D (2018). The effects of state-level pharmacist regulations on generic substitution of prescription drugs. Health Econ.

[5] Anis AH (2000). Pharmaceutical policies in Canada: another example of federal-provincial discord. CMAJ.

[6] Beecroft G (2007). Generic drug policy in Australia: a community pharmacy perspective. Australia and New Zealand Health Policy.

[7] Heikkilä R, Mäntyselkä P, Hartikainen-Herranen K, Ahonen R (2007). Customers’ and physicians’ opinions of and experiences with generic substitution during the first year in Finland. Health Policy.

[8] Babar Z-U-D, Stewart J, Reddy S, Alzaher W, Vareed P, Yacoub N (2010). An evaluation of consumers’ knowledge, perceptions and attitudes regarding generic medicines in Auckland. Pharm World Sci.

[9] Heikkilä R, Mäntyselkä P, Ahonen R (2011). Do people regard cheaper medicines effective? Population survey on public opinion of generic substitution in Finland. Pharmacoepidemiol Drug Saf.

[10] Kobayashi E, Karigome H, Sakurada T, Satoh N, Ueda S (2011). Patients’ attitudes towards generic drug substitution in Japan. Health Policy.

[11] Heikkilä R, Mäntyselkä P, Ahonen R (2012). Why people refuse generic substitution: a population survey of public opinion on generic substitution in Finland. Drugs Ther Perspect.

[12] Quintal C, Mendes P (2012). Underuse of generic medicines in Portugal: an empirical study on the perceptions and attitudes of patients and pharmacists. Health Policy.

[13] Drozdowska A, Hermanowski T (2015). Exploring the opinions and experiences of patients with generic substitution: a representative study of polish society. Int J Clin Pharm.

[14] Kjoenniksen I, Lindbaek M, Granas AG (2006). Patients’ attitudes towards and experiences of generic drug substitution in Norway. Pharm World Sci.

[15] Palagyi M, Lassanova M (2008). Patients attitudes towards experience with use of generics in Slovakia, performance of generic substitution. Bratisl Lek Listy.

[16] Shrank WH, Cadarette SM, Cox E, Fischer MA, Mehta J, Brookhart AM (2009). Is there a relationship between patient beliefs or communication about generic drugs and medication utilization?. Med Care.

[17] Colgan SLE, Faasse K, Pereira JA, Grey A, Petrie KJ (2016). Changing perceptions and efficacy of generic medicines: an intervention study. Health Psychol.

[18] Håkonsen H, Eilertsen M, Borge H, Toverud E-L (2009). Generic substitution: additional challenge for adherence in hypertensive patients?. Curr Med Res Opin.

[19] Gill L, Helkkula A, Cobelli N, White L (2010). How do customers and pharmacists experience generic substitution?. Int J Pharm Healthc Mark.

[20] Toverud E-L, Røise AK, Hogstad G, Wabø I (2011). Norwegian patients on generic antihypertensive drugs: a qualitative study of their own experiences. Eur J Clin Pharmacol.

[21] Skaltsas LN, Vasileiou KZ (2015). Patients’ perceptions of generic drugs in Greece. Health Policy.

[22] Hakonsen H, Toverud E-L (2011). Special challenges for drug adherence following generic substitution in Pakistani immigrants living in Norway. Eur J Clin Pharmacol.

[23] Chee Ping C, March G, Clark A, Gilbert A, Hassali MA, Bahari MB (2010). A web-based survey on Australian community pharmacists’ perceptions and practices of generic substitution. Journal of Generic Medicines.

[24] Alkhuzaee FS, Almalki HM, Attar AY, Althubiani SI, Almuallim WA, Cheema E (2016). Evaluating community pharmacists’ perspectives and practices concerning generic medicines substitution in Saudi Arabia: a cross-sectional study. Health Policy.

[25] Olsson E, Wallach-Kildemoes H, Ahmed B, Ingman P, Kaae S, Kälvemark SS (2017). The influence of generic substitution on the content of patient-pharmacist communication in Swedish community pharmacies. Int J Pharm Pract.

[26] Finnish Government. Government bill 165/2002 on amending the medicines act and the Health insurance act. 2002. https://www.eduskunta.fi/FI/vaski/HallituksenEsitys/Documents/he_165+2002.pdf accessed 2 Jun 2019. Finnish

[27] Finnish Government. Government Bill 100/2008 on amending the Health Insurance Act and the Medicines Act. 2008. https://www.eduskunta.fi/FI/vaski/HallituksenEsitys/Documents/he_100+2008.pdf . Accessed 2 Jun 2019. Finnish

[28] Finnish Government. Medicines Act 395/1987. 1987. http://www.finlex.fi/fi/laki/ajantasa/1987/19870395 . Accessed 2 Jun 2019. Finnish.

[29] Finnish Medicines Agency. Criteria used in compiling the list. 2018. https://www.fimea.fi/web/en/databases_and_registeries/substitutable_medicinal_products/criteria_used_in_compiling_the_list. Accessed 2 Jun 2019.

[30] Finnish Government. Health insurance act of 1224/2004. 2004. http://www.finlex.fi/fi/laki/ajantasa/2004/20041224. Accessed 2 Jun 2019. Finnish.

[31] Association of Finnish Pharmacies. Generic substitution at pharmacies has already generated savings of more than 1 billion euros. 2018. https://www.apteekkariliitto.fi/media/tiedotteet/apteekkien-toteuttamat-laakevaihdot-saastaneet-jo-yli-miljardia-euroa.html. Accessed 2 Jun.2019. Finnish.

[32] The Social Insurance Institution of Finland. Generic substitution and the reference price system. 2017. https://www.kela.fi/laakevaihto-ja-viitehintajarjestelma-2017. Accessed 2 Jun 2019 Finnish

[33] Finnish Government. Government Bill 330/2014 on amending the Health Insurance Act and section 57 of the Medicines Act. 2014. https://www.eduskunta.fi/FI/vaski/HallituksenEsitys/Documents/he_330+2014.pdf . Accessed 2 Jun 2019. Finnish.

[34] Finnish Government. Government Bill 184/2016 on amending and temporarily amending the Health Insurance Act and on amending sections 57 b and 102 of the Medicines Act and sections 22 and 23 of the Health Care Professionals Act. 2016. https://www.eduskunta.fi/FI/vaski/HallituksenEsitys/Documents/HE_184+2016.pdf . Accessed 2 Jun 2019. Finnish

[35] Finnish Government. Act 253/2015 on amending section 57 of the medicines act. 2015. https://www.finlex.fi/fi/laki/alkup/2015/20150253. Accessed 2 Jun 2019. Finnish.

[36] Finnish Government. Act 1100/2016 on amending and temporarily amending the Health Insurance Act. 2016. http://finlex.fi/fi/laki/alkup/2016/20161100 . Accessed 2 Jun 2019. Finnish.

[37] Association of Finnish Pharmacies. Pharmacies in numbers. 2017. https://www.apteekkariliitto.fi/apteekkitieto/apteekit-numeroina.html . Accessed 2 Jun 2019. Finnish.

[38] Finnish Medicines Agency Administrative Regulation No 2/2016: Medicine Dispensing 2/2016. 2016. https://www.fimea.fi/documents/160140/764653/20644_Maarays_laakkeiden_toimittamisesta_SUOMI_2011-12-19.pdf . Accessed 2 Jun 2019. Finnish

[39] Hartikainen-Herranen K, Ahonen R. Effects of generic substitution on pharmacies’ finances and operations. In: Ahonen R, Martikainen J, editors. The first year of generic substitution. Helsinki: the social insurance institution of Finland; 2005, p. 69–78. Finnish

[40] Timonen J, Kauppinen H, Ahonen R. Problems and development areas regarding electronic prescriptions a survey among pharmaceutical staff in pharmacies. Suomen Lääkärilehti. 2016:51–8. Finnish

[41] University of Eastern Finland: Instructions and forms of UEF Committee on Research Ethics. http://www.uef.fi/en/web/guest/research/instructions-and-forms (2019). Accessed 1 Jun 2019.

[42] Finnish Advisory Board on Research Integrity. Ethical principles of research in the humanities and social and behavioral sciences and proposals for ethical review 2009. https://www.tenk.fi/sites/tenk.fi/files/ethicalprinciples.pdf. Accessed 1 Jun 2019.

[43] Finnish Advisory Board on Research Integrity. Responsible conduct of research and procedures for handling allegations of misconduct in Finland 2012. 2012. https://www.tenk.fi/sites/tenk.fi/files/HTK_ohje_2012.pdf . Accessed 1 Jun 2019.

[44] Kobayashi E, Satoh N, Ueda S (2011). Community pharmacists’ perspectives on generic substitution in Japan. J Public Health.

[45] Heikkilä R, Mäntyselkä P, Ahonen R (2011). Price, familiarity, and availability determine the choice of drug a population-based survey five years after generic substitution was introduced in Finland. BMC Clin Pharmacol.

[46] Toklu HZ, Dülger GA, Hıdıroğlu S, Akici A, Yetim A, Gannemoğlu HM (2012). Knowledge and attitudes of the pharmacists, prescribers and patients towards generic drug use in Istanbul Turkey. Pharmacy practise.

[47] Yousefi N, Mehralian G, Peiravian F, NourMohammadi S (2015). Consumers’ perception of generic substitution in Iran. Int J Clin Pharm.

[48] Babar Z-U-D, Grover P, Stewart J, Hogg M, Short L, Seo HG (2011). Evaluating pharmacists’ views, knowledge, and perception regarding generic medicines in New Zealand. Res Social Adm Pharm.

[49] Olsson E, Kälvemark SS (2012). Pharmacists’ experiences and attitudes regarding generic drugs and generic substitution: two sides of the coin. Int J Pharm Pract.

[50] Maly J, Dosedel M, Kubena A, Vlcek J (2013). Analysis of pharmacists’ opinions, attitudes and experiences with generic drugs and generic substitution in the Czech Republic. Acta Pol Pharm.

[51] Mondelo-García C, Mendoza E, Movilla-Fernández M-J, Coronado C (2018). Perceptions of pharmacists and physicians on generic substitution in a financial crisis context in northwestern Spain: a qualitative study. Health Policy.

[52] O’Leary A, Usher C, Lynch M, Hall M, Hemeryk L, Spillane S (2015). Generic medicines and generic substitution: contrasting perspectives of stakeholders in Ireland. BMC Res Notes.

[53] Wong ZY, Hassali MA, Alrasheedy AA, Saleem F, Yahaya AH, Aljadhey H (2014). Patients’ beliefs about generic medicines in Malaysia. Pharmacy Practise.

[54] Himmel W, Simmenroth-Nayda A, Niebling W, Ledig T, Jansen R-D, Kochen MM (2005). What do primary care patients think about generic drugs?. Int J Clin Pharmacol Ther.

[55] Dunne S, Shannon B, Dunne C, Cullen W (2014). Patient perceptions of generic medicines: a mixed-methods study. Patient..

[56] Shrank WH, Cox ER, Fischer MA, Mehta J, Choudhry NK (2009). Patients’ perceptions of generic medications. Health Aff.

[57] Toverud E-L, Hartmann K, Håkonsen H (2015). A systematic review of physicians’ and pharmacists’ perspectives on generic drug use: what are the global challenges?. Appl Health Econ Health Policy.

