# Supplementary material for: The content of patient counseling about interchangeable medicines and generic substitution in Finnish community pharmacies - a survey of dispensers
Source: BMC Health Serv Res. 2019 Dec 11;19:956. doi: 10.1186/s12913-019-4798-2 (PMC6907127; doi:10.1186/s12913-019-4798-2)
Supplement: Supplementary file 1 — Additional file 1. Questionnaire - Survey for dispensers regarding generic substitution and reference price system. [file 12913_2019_4798_MOESM1_ESM.pdf]

University of Eastern Finland
Faculty of Health Sciences
School of Pharmacy
February 2018

UNIVERSITY OF EASTERN FINLAND

Survey for dispensers regarding generic substitution and reference price system

Answer the questions by circling the number of the appropriate response option. If necessary, write the answer in the space reserved. It is important to answer all the questions.

1. Are you working at a pharmacy at the moment?

1 No → Please return the blank form in the attached return envelope.

2 Yes → Please continue filling in the form.

2. Your year of birth? ________________

3. Your gender?

1 Male

2 Female

4. Number of prescriptions dispensed in 2017 at the pharmacy where you are working?

1 ≤30,000

2 30,001 to 60,000

3 60,001 to 100,000

4 ≥100,001

5. Location of the pharmacy where you are working?

1 Southern Finland

2 Southwestern Finland

3 Western and Inland Finland

4 Eastern Finland

5 Northern Finland

6 Lapland

6. What do you think about the following statements? In each statement, circle the number of the option you consider appropriate.

	Completely agree	Agree to some extent	Disagree to some extent	Completely disagree	Don't know
I think that the possibility to substitute a medicine at a pharmacy with an equivalent, but cheaper, medicinal product is a good thing	1	2	3	4	5
Generic substitution usually goes smoothly when dispensing prescriptions	1	2	3	4	5
The price advice obligation regarding the cheapest medicine is well observed at the point of dispensing the prescription	1	2	3	4	5
The reference price band is too narrow	1	2	3	4	5
Interchangeability of antiepileptics in indications other than epilepsy is a good reform	1	2	3	4	5
Generic substitution of antiepileptics is simple	1	2	3	4	5
The price advice obligation reduces the time used for other medication counselling	1	2	3	4	5
Inclusion of parallel-imported and parallel-distributed products in the scope of the reference price system is a good reform	1	2	3	4	5
Customers usually understand the principles of reimbursements by the Social Insurance Institution (Kela) in the reference price system	1	2	3	4	5

7. **What do you tell customers about <u>medicines included in the reference price system</u> when dispensing prescriptions? In each case, circle the number of the option you consider appropriate.**

	**Always**	**Often**	**Rarely**	**Only when asked**
That the customer can change the medicine for an equivalent, but cheaper, medicinal product	1	2	3	4
The medicine's inclusion status in the reference price band if it is outside the reference price band	1	2	3	4
The medicine's inclusion status in the reference price band if it is within the reference price band	1	2	3	4
Customers can choose their medicines from among several product alternatives	1	2	3	4
That there are price differences between interchangeable medicinal products	1	2	3	4
About reimbursability of the medicine by the Social Insurance Institution	1	2	3	4
That there are differences in composition between interchangeable medicinal products (if there are any, e.g. may contain lactose)	1	2	3	4
That there are differences between interchangeable medicinal products in terms of packaging (if there are any, e.g. container, blister)	1	2	3	4
That there are differences in the appearance of interchangeable medicinal products (if there are any, e.g. tablet shape, colour)	1	2	3	4
About availability of interchangeable medicinal products	1	2	3	4
Which pharmaceutical company has manufactured the medicinal product	1	2	3	4
That the customer has the right to decline generic substitution	1	2	3	4
That generic substitution has been prohibited by the physician if recorded in the prescription	1	2	3	4
How generic products differ from original products	1	2	3	4
How parallel-distributed or parallel-imported products differ from original products if they exist in the reference price group	1	2	3	4
About other matters. What are the most common ones? ____ ____				

8. **When you are dispensing medicines within the reference price system, do you tell the customer about the <u>least expensive</u> substitutable product at the point of dispensing?**
   1 Always → Go to question **10.**
   2 Often
   3 Rarely
   4 Only when asked

9. **In what situations do you not tell the customer about the least expensive substitutable product at the point of dispensing when you are dispensing medicines within the reference price system?**

____

____

____

10. **What are the most common questions asked by customers regarding generic substitution and the reference price system?**

____

____

____

11. **What things make it easier to give advice to customers about generic substitution and the reference price system?**

____

____

____

12. **What things make it more difficult to give advice to customers about generic substitution and the reference price system?**

____

____

____

13. **On what grounds do you choose the interchangeable medicinal product <u>primarily offered</u> to the customer <u>within the reference price group</u>?**

____

____

____

14. **On what grounds does your pharmacy choose which interchangeable prescription medicinal products to stock? You may choose several options.**

1 The product is in the reference price band
2 The product is the cheapest in the reference price band
3 The product is the most expensive in the reference price band
4 The product has a long shelf life
5 The product is manufactured by a reliable pharmaceutical company
6 The product is Finnish
7 The product is usually readily available
8 The product is supplied by a reliable pharmaceutical wholesaler
9 The pharmaceutical company compensates for the fall in stock value resulting from changes in the product's price
10 The pharmaceutical company compensates for expired medicinal products.
11 Other factor. Which?

____

12 Don't know

15. Are there any problems related to stocking the cheapest medicinal product in reference price groups?

1 No

2 Yes. What kind?

____

____

16. In your opinion, what are the main advantages of generic substitution and the reference price system?

____

____

____

____

17. In your opinion, what are the main problems with generic substitution and the reference price system?

____

____

____

____

18. Are the law and official regulations regarding generic substitution and the reference price system clear?

1 Yes

2 Don't know

3 No. What things are problematic? ____

____

____

Here you can write your comments about this survey and your experiences of generic substitution and the reference price system. If necessary, you can continue e.g. on the back of the cover letter and return it with the questionnaire.

____

____

____

____

____

____

____

____

____

____

Thank you!
